# 

**Published:** 1890-11

**Authors:** 


Whole Number, CCCLII.

New Series.

**November, 1890.**

VOL. XXX. No. 4.

### BUFFALO MEDICAL AND SURGICAL JOURNAL.

Established 1845 by AUSTIN FLINT, M. D.

**Editors:**

THOS. LOTHROP, M. D. WM. WARREN POTTER, M. D.

**Associate Editors:**

FRANK HAMILTON POTTER, M. D. JAMES WRIGHT PUTNAM, M. D.

WILLIAM C. KRAUSS, M. D. JOHN PARMENTER, M. D.

JOHN A. MILLER, Ph. D. DeLANCEY ROCHESTER, M. D.

SCRIPTA FERUNT ANNOS

European Agency:
TRÜBNER & CO.,
57 and 59 Ludgate Hill, London, E. C.

BUFFALO:
1890.

Foreign Subscription, $2.50

Entered at the Post-Office at Buffalo, N. Y., as Second-class Mail Matter.

*Times Printing House, Buffalo, N. Y.*

SUBSCRIPTION PRICE, $2.00 IF PAID IN ADVANCE; OTHERWISE $2.50 A YEAR.

PUBLISHED MONTHLY.

Table of Contents on Page V.

